# Medical and Physical Intelligence

**Published:** 1823-10

**Authors:** 


					MEDICAL AND PHYSICAL INTELLIGENCE.
MORBID ANATOMY.
1. Monstrous Crania.?Cuvier lately read a memoir before the
members of the Institute, on the subject of two heads of enormous size,
which had become the source of a thousand ridiculous stories. The
first was found in Germany, and has long been regarded by various
writers as having belonged to a race of giants, who had disappeared
from the earth at the period of the deluge. Soemmering, after an
attentive examination, was led to suppose tlie great development of this
head as arising from a diseased action. Cuvier has continued this opi-
nion, and maintains, besides, that not only is the head in question one
which belonged to an individual of the ordinary race, but that it must
have been the head of a child, in which the enlargement took place
prior to the development of the teeth, having discovered several of these
similar to the milk-teeth of infants. The second wonder is a Parisian
skull found in the environs of the town, and preserved in the museum of
M. dk Jussieu. This presents no very remarkable external deformity,
and the size does not seem considerably augmented; but the weight is
extraordinary, and the thickness one inch and a half: it has acquired the
hardness of ivory. This morbid organization of bone has been observed
frequently in other parts: the thickness, hardness, and weight of the
cranium, generally depend upon an altered state of the brain.?(Revue
Mcdicale, Juillet *1823.)
4
Medical and Physical Intelligence. 351
PATHOLOGY.
2. On the Seat of Extravasation in Hemiplegia.?Messrs. Feville
and Pinel-Grandcham ps give the following account of an old
Woman, who died at the Salpetriere. She had been affected for many
>ears with complete paralysis of the upper and lower extremities of the
leftside. The remains were found of an old extravasation of blood in
the right hemisphere of the brain, in the medullary space between the
?plic thalami and the corpora striata, involving both these parts equally.
Messrs. Feville and Pinel-Grandchamps laid this case before the Aca-
demy of Medicine, as tending to confirm their former observation on
the seat of movement in both members. They are of opinion that the
sent of motion of the upper extremity is situated in the optic thalami,
and that of the inferior extremity in the corpora striata. Iti a case
where the arm and leg are both struck with palsy, the optic thalami and
corpora striata must, according to their views, be both alike disorga-
nized. In the Number of MajenDIe's Journal for April, M.Seurks
like wise maintains the opinion that the cause of ihe movements of the
upper extremities resides in the posterior part of the hemispheres, and
the cause of movement of the lower limbs in the anteiior part of the
brain.?(Revue Medicate, Juiilet.)
3. Hernia through the Diaphragm.?M. Jules Cloouet has re-
lated a case of this nature, of recent occurrence. The subject of his
observation was a man of about forty-live years of age, of healthy ap-
pearance, who had the thorax strongly compressed from before back-
wards, between the wheels of two carriages. The patient, who was
carried to the hospital of St. Louis immediately after the accident, com-
plained of intolerable pain in the chest. The respiration was difficult
and interrupted ; the pulse accelerated and intermitting; the face swol-
len, of a purple colour, and having a very peculiar expression of pain.
Antiphlogistic remedies were employed in their fullest extent: never-
theless the patient died thirty-six hours after the accident had occurred,
^n examining the body, the soft parietes of the thorax presented no
lesion, but several ribs were fractured; the diaphragm was considerably
!?rn at the left side, the rupture extending towards the centre; the pe-
ricardium was lacerated in all its anterior and inferior portion; the whole
the stomach, and the greater part of the colon, hail passed into the
left cavity of the chest, and was situated in immediate contact with the
1'^art and lung: this last was pressed back upon the vertebral column.
Ihe cavity of the pleura was filled with blood.?(Ibid.)
4. Blue-coloured Urine.-The same gentleman relates an instance
a hoy of thirteen \ears old, who, during three successive oays, in the
course of a severe attack of enteritis, voided urine of a pure blue colour,
|?aded with sediment of the same appearance, which communicated a
be?uiiful indigo tint to paper. One of the members of the Academy,
Vyho was present during the relation of this case, mentioned ins having
once seen a similar phenomenon in the person of a man affected w ith
a?ulc rheumatism.-(Ibid.)
352 Medical and Physical Intelligence.
SURGERY.
5. Swelling of the Tongue cured by Incisions.?A woman, aged
twenty.eight years, was exposed to cold and anxiety of mind during
menstruation, by one or both of which means the discharge was suddenly
much diminished. To this supervened a violent affection of the tongue,
which impeded deglutition so much as to induce her attendant to order
the application of fifteen leeches to the labia, bleeding from the foot,
and an irritating enema. Notwithstanding these remedies, the tongue
became so much swelled as to press backwards into the pharynx, and
backwards out of the mouth between the teeth: suffocation, under these
circumstances, became imminent. Respiration could only be performed
through the nose; tlie evacuation of the sputa was extremely difficult.
Thirty leeches on the neck produced only very temporary relief; when
the surgeon (M. Faneac de la Cgur) made two deep incisions from
the root lo the point of the tongue, after the manner recommended by
JDe Lamalle, in his memoir presented to the Royal Academy of
Surgery, in 1752, and by Job-a-Meekren, a Dutch surgeon, who
hud practised the operation in 1656. Considerable diminution of the
tongue resulted, which however remained much larger than in its natu-
ral state. An emetic of tartarized antimony completed the cure. It is
worthy of remark that, in some of these instances of violent swelling of
the tongue, the disease only affects one side.?(Bulletin de la Socitta
Medicate d'Indre-et.Loire.)
CHEMISTRY.
6. TJrte in the Blood.?MM. Prevost and Dumas, of Geneva,
liave published an account of some experiments 011 tlie blood, which
formed the subject of a memoir read before the Society of Physics and
Natural History of that town, in 1821. On a future occasion we in-
tend to give a more detailed account of this paper: at present we shall
only mention, that the kidneys of various animals were removed, and
the blood subjected to careful analyses, when a considerable portion of
uree was discovered, which differed but very slightly from that found
in the urine of some animals. " The physiologists (say the authors,)
who may be curious to satisfy themselves of the truth of the facts
mentioned in this memoir, will not experience much difficulty. Five
ounces of the blood of a dog, which has lived without kidneys for only
two days, afford more than twenty grains of uree; and two ounces
of the blood of a cat, under the same circumstances, give more than teu
grains. These quantities are perfectly appreciable by the least experi-
enced chemists. We would not advise them to try on rabbits, because
they do not resist the operation so well." M.Vauquelin, whose
name ranks so high as a chemist, repeated these investigations along
with M. Segalas, and concludes from them that uree acts as a very
active diuretic.?(Annates de Cliimie, fyc. Mai.)
MISCELLANEOUS.
7. Artificial Palate.?The natural want or the casual destruction of
that delicate organ, the human palate, is attended with the most un-
Medical and Physical Intelligence. 353
pleasant of all effects?the loss of voice; and, of the many substitutes
which we have, verv few have those advantages which could be wished.
The common metallic palate seldom fits well, and always gives pain;
while those of gum caoutchouc and other elastic substances are ollensive,
and also, by pressing asunder the parts, increase the deficiency. The
removal of them all, for the purpose of cleaning, is a work of some
trouble. We have seen a silver palate, constructed by Mr. Clark, of
Grosvenor-street, a very ingenious dentist, which obviates many of the
objections to the old construction. It fits the parts with the utmost
nicety, and, as it does not at all press upon the edges of the deficiency,
allows the parts to contract, or even to be to a certain extent repro-
duced ; while the wearer can take it out, clean it, and replace it, in two
?r three minutes. When it is to be removed or put in, the wings which
fasten it to the upper side are made to collapse into a very small space ;
and, after it is put in its place, they are made to expand aud embrace
the edges of the bone, with any degree of tightness that may be neces-
sary. The whole of the machinery (which is very neat,) is worked by
a small button in the centre of the palate, so flat as to give no uneasi-
ness to the tongue, and yet which can be moved with the greatest ease.
Besides the facility with which this palate can be removed and replaced,
the great advantage of it consists in the accuracy with which it fits the
parts. The inventor, being an expert worker in metals, cuts and
Works the whole himself; and by this means is enabled to procure
a perfect model, and also fit it precisely. In some very bad cases, Mr.
Clark has fitted those palates with joints, by which they could, without
any pain to the wearer, be made fast to the moveable parts of the
"louth. it gives us pleasure to notice this invention, not more on ac-
count of the merit of the inventor, than of his modesty. It is four or
five years since Mr. Clark made and fitted the first palate of this kind ;
surgeons of the first consequence in town, and also in Edinburgh, have
spoken highly of it; and yet the inventor has not, in the usual way,
called the attention and claimed the patronage of the public.
[ 354 J
METEO110LOGICAL JOURNAL,
From August 20, to September 19, 1823.
By Messrs. William Harris and Co. 50, Holborn, London.
A hi
'20
*? o
22
23
21
2.)
2 6
27
28
29
30
31
Sep.
1
2
3
4
5
<*)
7
3
9
to
11
12
1 j
14
to
16
17
1!!
19
llain
iiiuge
.05
.12
74 54
70153
29.65
29.64
29.62
29.50
29.67
29.63
29.80
29.90
30.00
29.75
29.80
29.93
30.02
29.87
29.87
29.96
29.93
29.97
29.97
30.04
30,00
30.00
30.06
29.87
29.68
29.62
29.18
29.62
29.70
30.10
30.10
29.65
29.72
29.50
29.60
29.67
29.67
29.80
29.83
29.88
29.77
29.80
30.02
29.96
29.80
29.93
30 00
29.93
29.90
30.00
30.02
29.98
30.00
30.04
29.76
29.68
29.42
29.52
29.74
29.84
30.13
29.97
DE
LUC'S
IIYG.
9 A.M.
s w
S W
SSE
ssw
SSE
SSE
N
NE
SSW
sw
SSW
SSW
sw
sw
sw
sw
wsw
WNW
NNE
NNE
NNE
NNE
E
E
S
S
SW
SSW
SSW
w
NNW
10P.M
S
SSW
SSW
sw
s
wsw
NE
SSW
SW
SSW
wsw
wsw
wsw
sw
w
sw
w
N
NNE
NE
NNE
NE
E
SSE
SSW
SE
SSW
S
w
NNW
S
atmospheric
V A UI VTION.
9 A.M.
Fine
Rain
Cloud.
Slio'ry
Cloud.
Fme
Cloud.
Fme
Fine
Foggy
Fine
Cloud
Cloud.
Storm.
Fine
Cloud.
Fine
Foggy
2 P.M.
Fine
Rain
Rain
Very F
Rain
Fine
Very l<
Fine
Rain
Fine
10p.m.
Fine
Rain
Fine
Rain
Fine
Rain
Very F
Fine
Fine
The quantity of rain fallen in the month of August,
was 1 inch and 77.1 OOtiis.

				

